# Pulmonary Artery Thromboembolism (PATE) in Paediatrics: Three Clinical Case Presentations

**DOI:** 10.15388/Amed.2026.33.1.24

**Published:** 2026-07-22

**Authors:** Gabija Asipauskaitė, Greta Miškinaitė, Algirdas Dagys, Vaidotas Gurskis

**Affiliations:** 1 Medical Academy, Faculty of Medicine, Lithuanian University of Health Sciences, Kaunas, Lithuania.; 2 Department of Paediatric Emergency Medicine, Lithuanian University of Health Sciences Kaunas Clinics, Kaunas, Lithuania.; 3 Paediatric Intensive Care Unit, Lithuanian University of Health Sciences Kaunas Clinics, Kaunas, Lithuania.

**Keywords:** Pulmonary embolism, pulmonary artery thromboembolism, paediatrics, children, plaučių embolija, plaučių arterijos tromboembolija, pediatrija, vaikai

## Abstract

**Background:**

Pulmonary artery thromboembolism in children is an exceptionally rare condition. Since its clinical presentation and associated risk factors differ from those observed in adults, the condition is frequently underdiagnosed.

**Case description:**

We present three cases of paediatric pulmonary artery thromboembolism, each with distinct clinical presentations and differing risk factors. The first case involved a 15-year-old boy who developed fatal massive bilateral pulmonary artery thromboembolism after having spinal trauma, surgery, immobility, and central venous catheter placement. The second case concerned a 17-year-old girl with Prader–Willi syndrome and multiple comorbidities, who presented with severe respiratory failure and laboratory evidence suggesting pulmonary thromboembolism, although imaging to confirm the diagnosis was limited. The third case involved a previously healthy 17-year-old boy with upper-extremity deep venous thrombosis and confirmed pulmonary artery thromboembolism despite the absence of identifiable provoking factors.

**Conclusions:**

These cases illustrate the heterogeneity of paediatric PATE presentation, the variability of the underlying risk factors, and the diagnostic challenges encountered in clinical practice, underscoring the need for heightened awareness among clinicians.

## Abstract (it)

**Įvadas:**

Plaučių embolija pediatrijoje yra ypač reta būklė. Klinikinė išraiška ir rizikos veiksniai dažnai skiriasi nuo suaugusiųjų, todėl klinikinėje praktikoje ši būklė dažnai lieka nediagnozuota.

**Atvejų aprašymas:**

Pateikiame tris vaikų plaučių embolijos atvejus, pasižyminčius skirtinga klinikine išraiška ir įvairiais rizikos veiksniais. Pirmas aprašomas atvejis – 15 metų vaikinui po stuburo traumos, operacijos, ilgalaikio nejudrumo ir centrinės venos kateterizacijos išsivystė masyvi abipusė plaučių arterijų tromboembolija, pasibaigusi mirtimi. Antras aprašomas atvejis – 17 metų mergina, serganti Prader-Willi (PWS) sindromu ir turinti kelias gretutines patologijas. Šiai pacientei pasireiškė sunkus kvėpavimo nepakankamumas, o laboratoriniai rodmenys kėlė įtarimą dėl plaučių embolijos, nors vaizdinės diagnostikos galimybės diagnozei patvirtinti buvo ribotos. Trečias atvejis – anksčiau buvęs sveikas 17 metų vaikinas, kuriam buvo nustatyta giliųjų venų trombozė ir patvirtinta plaučių arterijų embolija, aiškaus trombozę sukeliančio veiksnio nebuvo nustatyta.

**Išvados:**

Šie atvejai parodo vaikų plaučių arterijų embolijos klinikinės išraiškos įvairovę, skirtingus rizikos veiksnius ir klinikinėje praktikoje kylančius diagnostinius sunkumus, siekiant atkreipti gydytojų dėmesį į šią ligą.

## Introduction

*Pulmonary Artery Thromboembolism* (PATE) is the blockage of a pulmonary artery or its branches by a thrombus. PATE in paediatric population is considered rare with an incidence rate of 3.5–6 pulmonary artery thromboembolism-related hospitalisations per 100,000 people [1,2]. In recent years, the number of children diagnosed with pulmonary artery thromboembolism has been rising. This trend is thought to reflect an improved survival among those with chronic diseases, along with a more frequent use of central venous catheters and interventional procedures [3]. Between 79% and 98% of paediatric patients with pulmonary artery thromboembolism have at least one identifiable risk factor, with the most common one being the presence of a central venous catheter, obesity, congenital heart disease and other hypercoagulable conditions [4,5]. In children, PATE may present with pleuritic chest pain, dyspnoea, and signs of *Deep Vein Thrombosis* (DVT). However, this presentation is often incomplete or entirely absent. Even large emboli may be well compensated because children have a strong cardiopulmonary reserve. As a result, paediatric PATE may present only with subtle, non-specific symptoms, and can easily mimic other pulmonary conditions [5,6]. Consequently, diagnosis is frequently delayed [7,8]. Autopsy studies indicate that pulmonary artery thromboembolism was included in the initial differential diagnosis in only 15% of paediatric patients who ultimately died from the condition [7]. Therefore, it is crucial to raise awareness of pulmonary artery thromboembolism in children. We present three clinical cases aimed at highlighting the nonspecific nature of PATE symptoms, the associated risk factors, and the diagnostic challenges encountered in the paediatric population.

## Case presentations

### Case no. 1

A 15-year-old boy was brought to the Emergency Department with a spinal injury after an unsuccessful dive into water. He was admitted to the Paediatric Intensive Care Unit. The initial laboratory tests showed: aPTT 39.3 s, D-dimers 0.99 mg/L, fibrinogen 2.56 g/L, PT 26 s, PT activity 78% (all within the normal range). Spinal surgery was performed under general anaesthesia. Postoperatively, the patient’s condition was stable. He was able to move his upper limbs but had no motor function in the lower limbs. Clinical examination confirmed paraplegia with complete loss of sensation. Physiotherapy was initiated. Additionally, on the first day of hospitalization, a *Central Venous Catheter* (CVC) was placed, and thrombosis prophylaxis with *Fraxiparine* 0.3 ml once daily was initiated.

After 2 days, the patient developed fever and was started on antibiotic treatment. Inflammatory markers decreased. However, after one week of treatment, fever recurred. There was no bacteriological growth in blood and urine cultures, and chest radiography showed no signs of pulmonary infection. No pressure ulcers were present. Antibacterial therapy was adjusted. During this period, the patient’s clinical condition appeared to improve. Throughout the entire course of treatment, the patient was breathing spontaneously and did not require supplemental oxygen.

On the 16^th^ day of hospitalization, the patient suddenly complained of severe headache and immediately lost consciousness. His pulse became irregular, followed by cardiac arrest. Cardiopulmonary resuscitation was started following the asystole algorithm.

Bedside echocardiography performed during resuscitation revealed a markedly dilated right ventricle with freely floating hyperechogenic masses consistent with thrombi. A chest X-ray showed a mildly increased cardiac diameter. Thrombolysis was performed. Despite ongoing resuscitation efforts, return of spontaneous circulation was not achieved, and the patient was pronounced dead 2 hours later. An autopsy revealed extensive deep vein thrombosis of the lower limbs and acute massive bilateral pulmonary artery thromboembolism.

### Case no. 2

A 17-year-old girl with a known history of Prader–Willi syndrome, morbid obesity (175 kg), *Arterial Hypertension* (AH), type 2 *Diabetes Mellitus* (DM) and sleep apnoea presented to the Emergency Department of a secondary-level hospital with acute expiratory dyspnoea, cyanosis of the lips, intermittent cough, and a recent fever peaking at 38.1°C. On clinical examination, the patient was lethargic with signs of respiratory distress, RR 50 breaths per minute, SpO2 60% (increased to 75% on a non-rebreather mask at 15 l/min), and marked use of accessory respiratory muscles. Auscultation revealed coarse bilateral breath sounds with expiratory wheeze and fine crackles, more prominent on the right side.

Laboratory data indicated infection, and a nasopharyngeal culture identified *Staphylococcus aureus* as the causative pathogen. Arterial blood gas analysis showed respiratory acidosis. Chest radiography revealed bilateral pleural effusions with features consistent with pleuropneumonia. D-dimer (10 559 mg/l), TnI (290 ng/l), and NT-pro BNP (3 879 pg/mL) were markedly elevated, which are findings suggestive of a prothrombotic process and myocardial injury. Bilateral lower extremity ultrasonography was performed, revealing no evidence of *Deep Vein Thrombosis* (DVT). Echocardiography revealed grade I pulmonary hypertension, whereas follow-up studies showed progressive cardiac chamber dilation. Although PATE was suspected clinically, no echocardiographic or other definitive evidence of acute pulmonary artery thromboembolism was identified, apart from elevated D-dimer levels. CT pulmonary angiography was indicated but could not be performed because the patient’s body habitus exceeded the scanner’s physical limitations. Overall, the picture was most consistent with cardiopulmonary failure, secondary to severe pleuropneumonia with suspected PATE.

Given the concern for PATE, subcutaneous Fraxiparine 0.9 ml was administered twice daily. Over the course of intensive care, D-dimer levels decreased over time (10 559 → 556 mg/L), TnI and NT-pro BNP normalized as well, and follow-up echocardiography showed no further progression of cardiac chamber dilation. Clinically, the patient showed improvement: dyspnoea resolved, the respiratory distress eased, the patient became alert and no longer dyspnoeic at rest. After 30 days in the intensive care unit, anticoagulation from Fraxiparine was transitioned to apixaban, and the patient was subsequently transferred to a tertiary care hospital for continued care.

### Case no. 3

A 17-year-old boy presented to the Emergency Department with swelling and bluish discoloration of the left upper limb. He reported pain in the axillary and supraclavicular area. The symptoms developed shortly after participating in a sports competition. He denied trauma, fever, or other systemic symptoms. An outpatient Doppler ultrasound performed prior to admission demonstrated acute thrombosis of the left subclavian vein and approximately 50% occlusion of the basilic vein, with a narrow band of residual blood flow around the thrombus. Several markedly enlarged lymph nodes were noted in the left axillary region. Objectively, all vital signs were within normal limits, the left arm was slightly paler, and the left armpit was painful to the touch. Laboratory tests showed mildly elevated a PTT of 40.4 s (reference range 28–38 s) and a fibrinogen level of 4.5 g/L (reference range 2–4 g/L), while D-dimer levels remained within the normal range at 0.79 mg/L.

Given the confirmed upper-extremity venous thrombosis and the elevated risk of pulmonary artery thromboembolism, a contrast-enhanced CT scan was performed. Bilateral central contrast-filling defects were identified in the segmental and subsegmental pulmonary arteries which confirmed acute pulmonary artery thromboembolism without signs of right ventricular overload. Treatment with unfractionated heparin was initiated, with dosing titrated according to a PTT. Throughout hospitalization, hemodynamics remained stable, and there was no need for supplemental oxygen. On follow-up vascular ultrasound, the thrombi were decreasing in size.

No provoking risk factors were identified, therefore, comprehensive genetic testing for thrombophilia was performed. The results showed that no prothrombin (F2) gene mutation or the Leiden factor (F5) mutation was detected. After 10 days of inpatient treatment, with symptomatic and clinical improvement, the patient was discharged for outpatient treatment with oral anticoagulants and follow-up after 2–3 months.

**Table 1:** Clinical characteristics of the cases

	Case no. 1	Case no. 2	Case no. 3
**Age**	15	17	17
**Sex**	Male	Female	Male
**Respiratory symptoms**	None	Tachycardia, cyanosis, dyspnoea.	None
**Risk Factors**	Central venous catheter, paraplegia, surgery, infection	Prader-Willi syndrome (morbid obesity, AH, DM and sleep apnoea syndrome), infection	–
**D-dimers (mg/L)** *Reference range 0 – 500 mg/L*	0.99 (taken 2 weeks prior embolism suspected)	10 559	0.79
**TnI (ng/L)** *Reference range 0 – 23 ng/L*	–	290	–
**NT-pro BNP (pg/mL)** *Reference range 0 – 125 pg/L*	–	3 879	–
**CT scan**	–	–	Acute pulmonary embolism without signs of right ventricular overload
**DVT**	Not suspected, but was found during the autopsy	No	Yes
**Family history**	None	None	Grandmother had Pulmonary embolism
**Genetic disorders**	–	Prader-Willi	None
**Final outcome**	Died	After recovery from PATE, the patient was transferred to a tertiary hospital for further management	Recovered completely

## Discussion

PATE in children is uncommon and rarely fatal [8]. The clinical presentation of paediatric PATE frequently overlaps with many common paediatric diseases, leading to an average diagnostic delay of one week after the symptom onset [5,7,9]. Therefore, compound adverse events within 90 days remain a significant clinical concern [10].

Classic symptoms, when present, include an increased shortness of breath, pleuritic chest pain, haemoptysis, cough, and even syncope. Patients may present with tachycardia, tachypnoea, and oedema due to deep vein thrombosis (DVT) [7]. Paediatric patients with PATE exhibit DVT in approximately 60% to 72.1% of cases, and up to 20% of venous thromboemboli in children arise from upper-extremity DVT, unlike in adults, where lower-extremity sources are most common [5,8].

Diagnosis of PATE in children is challenging not only because the clinical presentation can be misleading, but also because adult clinical prediction rules, such as Wells clinical score and Geneva score, have not been validated in paediatrics [11]. This limitation reflects developmental differences in haemostasis specific to children, including a reduced thrombin-generating capacity due to lower plasma prothrombin, higher concentrations of thrombin inhibitors such as alpha-2-macroglobulin, and distinct platelet–vessel-wall interactions [5]. Furthermore, the diagnostic utility of D-dimer for pulmonary artery thromboembolism in paediatric patients has not been fully validated, and elevated D-dimer levels are not considered a statistically significant risk factor for PATE in children [12,13]. The D-dimer therefore has low diagnostic utility in this population: it is normal in 15% to 40% of children, and, although 85% of children with PATE present with an elevated D-dimer, this finding lacks specificity and is not discriminatory for the diagnosis [5,14]. D-dimer levels can be higher when the clinical presentation is more pronounced. In the presented cases, one patient exhibited markedly elevated D-dimer levels, corresponding with a typical and prominent presentation of PATE. In contrast, in the other two cases, D-dimer levels remained within the normal range, despite the presence of acute PATE in both patients. Similarly, ECG findings in children with PATE are generally non-specific, with sinus tachycardia observed in around half of the cases, right axis deviation in about 10–15%, and the S1Q3T3 pattern being rare, thus highlighting that ECG alone is insufficient for diagnosis [5]. Imaging plays a key role in PATE diagnosis, with CT scans showing 89% sensitivity and 94% specificity when performed in patients with two or more risk factors [15]. Two paediatric studies reported high diagnostic accuracy for PATE using computed tomography pulmonary angiography (CTPA) performed with multislice spiral CT, which allows visualization of pulmonary arteries up to sixth-order branches, evaluation of mediastinal and parenchymal structures, and direct thrombus detection [16–18].

Magnetic resonance pulmonary angiography (MRPA) allows radiation-free evaluation of the pulmonary arteries and enables imaging of the upper body and central venous system in the same investigation, making it useful for patients with central venous lines or iodine contrast allergy [8]. However, its use is limited by long scan times, limited availability, monitoring challenges in critically ill children, and the need for general anaesthesia in younger patients [8,19]. Echocardiography, via transthoracic or transoesophageal views, can directly visualize thrombi in the heart and central pulmonary arteries, and reveal indirect signs of PATE such as right ventricular dilatation, septal motion abnormalities, tricuspid regurgitation, and persistent inferior vena cava diameter [20]. While not a routine diagnostic test, it is useful in critically ill patients to help differentiate massive PATE from other causes of hemodynamic instability. In our cases, echocardiographic findings correlated with the disease severity: Case No. 1 showed right ventricular dilatation and intracardiac thrombi, while Case No. 2 demonstrated pulmonary hypertension and chamber dilatation. Echocardiography was not performed in Case No. 3.

There are numerous risk factors for paediatric pulmonary embolism. The most significant risk factor contributing to the development of pulmonary embolism in the paediatric population is the *Central Venous Line* (CVL), which is becoming increasingly common in the paediatric practice for administering medications, parenteral nutrition or chemotherapy [8,13]. Implantation of CVLs can cause formation of non-adherent fibrin sleeves that may obstruct catheter tips. In addition, the catheter itself and infused agents (e.g., chemotherapy) can disrupt the normal blood flow, injure the local endothel of the vessel wall, and can create proinflammatory response to a foreign intravascular body, which also contributes to thrombus formation [8]. Spinal cord injury is among the strongest recognized risk factors for paediatric *Venous Thromboembolism* (VTE) because of prolonged immobility, and immobilization. Children with spinal cord injury have a 37-times higher risk of VTE compared to children without this injury [4]. Moreover, surgery itself is known to predispose children to PATE, and this risk increases further with a prolonged surgical duration [21]. The literature suggests that infection can also significantly increase the risk of thrombosis. The systemic inflammation associated with either acute or chronic infection can strongly promote coagulation factor activation [22].

One of our presented patients exhibited a particular set of risk factors related to her underlying genetic disorder. Prader–Willi syndrome (PWS) is an imprinting defect caused by the lack of expression of paternally inherited genes in the 15q11.2–q13 chromosomal region [23]. The syndrome itself is associated with multiple complications that are recognized contributors to thromboembolic risk. Obesity represents the strongest prothrombotic trigger, as it drives chronic inflammation, impairs fibrinolysis, and elevates fibrinogen, von Willebrand factor, and factor VIII levels [24]. Moreover, studies have shown that individuals with Prader–Willi syndrome and obesity have nearly a 5-fold higher risk of pulmonary embolism or DVT compared with obese individuals without the syndrome [23]. Additional comorbidities frequently observed in individuals with PWS, such as diabetes and hypertension, further promote a hypercoagulable state and sustain chronic systemic inflammation. Hypoxemia resulting from obstructive sleep apnoea also directly activates coagulation pathways, thereby compounding the patient’s overall thrombotic risk [23,25]. Taken together, these overlapping risk factors created a markedly hypercoagulable state, which likely played a crucial role in the development of the patient’s pulmonary embolism even at a young age.

Paediatric patients with PATE may also have other risk factors that contribute to a hypercoagulable state, such as congenital heart disease, thrombophilia, previous VTE, nephrotic syndrome, hormonal supplementation, or deep vein thrombosis [7–9,13]. Nevertheless, idiopathic thrombosis may still occur, although it is rare in the paediatric population. It is estimated that only 0–8% of pulmonary embolism cases in children are spontaneous, compared with adults, in whom, idiopathic events account for approximately 30% of the cases [8,12]. One of our presented patients had none of the known risk factors for DVT of the upper limb and PATE. With no causative factors found, the case could be considered idiopathic. However, considering the symptoms and the excluded risk factors, the patient’s presentation resembles *Paget–Schroetter Syndrome* (PSS). This syndrome is a rare primary deep vein thrombosis involving the axillary and subclavian veins. Patients with this condition are typically young and otherwise healthy, and the development of thrombosis is associated with compression of the subclavian vein in the costoclavicular space [26]. However, the diagnosis of PSS was not confirmed because no anatomical compression of the subclavian vein or other definitive diagnostic features were identified during evaluation.

The treatment of PATE in children largely follows the principles established in the adult practice, although evidence in children remains limited, and high-quality randomized trials are lacking. Moreover, there are limited data regarding differences between adult and paediatric PATE management because of the rarity of this condition in children [3]. Currently, the management of pulmonary thromboembolism in children includes several modalities, such as supportive care, anticoagulation with unfractionated heparin (UFH) or low molecular weight heparin (LMWH), vitamin K antagonists, systemic thrombolysis, placement of IVC filters, and mechanical or surgical thrombectomy. The optimal strategy is individualised based on the patient’s individual condition [3,8].

## Conclusions

Our case series highlights that pulmonary embolism in children is a rare but clinically important condition, frequently presenting with nonspecific symptoms. Importantly, the risk factors in paediatric patients differ from those seen in adults, thus necessitating a high index of suspicion and careful clinical assessment. An early recognition and timely diagnosis are crucial, as they may be life-saving. The cases presented here illustrate the wide variability in clinical presentation and diagnostic pathways, highlighting the need for heightened awareness. Given the limited paediatric evidence available, further research is essential to standardize diagnostic strategies and to develop management approaches tailored to the paediatric population.

## References

[1] Wolf S, Valerio L, Kucher N, Konstantinides SV, Klaassen ILM, van Ommen CH (2025). Acute pulmonary embolism in children and adolescents in the USA (2016 and 2019): a nationwide retrospective cohort study. *Lancet Respir Med*.

[2] Carpenter SL, Richardson T, Hall M (2018). Increasing rate of pulmonary embolism diagnosed in hospitalized children in the United States from 2001 to 2014. *Blood Adv*.

[3] Takahashi EA, Sista AK, Addison D, Bikdeli B, Bishay VL, Gu S (2025). Disparities in Current Pulmonary Embolism Management and Outcomes: A Scientific Statement From the American Heart Association. *Circulation*.

[4] Pelland-Marcotte M, Tucker C, Klaassen A, Avila ML, Amid A, Amiri N (2019). Outcomes and risk factors of massive and submassive pulmonary embolism in children: a retrospective cohort study. *Lancet Haematol*.

[5] Maggio A, Altieri L, Pantaleo D, Grignani M, Decembrino L (2022). Pulmonary embolism in children, a real challenge for the pediatrician: a case report and review of the literature. *Acta Biomed*.

[6] Biss TT, Brandão LR, Kahr WH, Chan AK, Williams S (2008). Clinical features and outcome of pulmonary embolism in children. *Br J Haematol*.

[7] Zaidi AU, Hutchins KK, Rajpurkar M (2017). Pulmonary Embolism in Children. *Front Pediatr*.

[8] Dijk FN, Curtin J, Lord D, Fitzgerald DA (2012). Pulmonary Embolism in Children. *Paediatr Respir Rev*.

[9] Tiratrakoonseree T, Charoenpichitnun S, Natesirinilkul R, Songthawee N, Komvilaisak P, Pongphitcha P (2024). Clinical prediction tool to identify children at risk of pulmonary embolism. *Thromb Res*.

[10] Lin ZL, Zeng SZ, Liu FQ, Shen T, Lyu Z, Zhang L (2025). Prognosis and influencing factors of pulmonary embolism in children: a multicenter study. *World J Pediatr*.

[11] Stein PD, Fowler SE, Goodman LR, Gottschalk A, Hales CA, Hull RD (2006). Multidetector computed tomography for acute pulmonary embolism. *N Engl J Med*.

[12] Sharaf N, Sharaf VB, Mace SE, Nowacki AS, Stoller JK, Carl JC (2018). D-dimer in Adolescent Pulmonary Embolism. *Acad Emerg Med*.

[13] Degerstedt SG, Winant AJ, Lee EY (2022). Pediatric Pulmonary Embolism: Imaging Guidelines and Recommendations. *Radiol Clin North Am*.

[14] Rajpurkar M, Biss T, Amankwah EK, Martinez D, Williams S, Van Ommen CH (2017). Pulmonary embolism and in situ pulmonary artery thrombosis in paediatrics. A systematic review. *Thromb Haemost*.

[15] Lee EY, Tse SKS, Zurakowski D, Johnson VM, Lee NJ, Tracy DA (2012). Children suspected of having pulmonary embolism: multidetector CT pulmonary angiography--thromboembolic risk factors and implications for appropriate use. *Radiology*.

[16] Kritsaneepaiboon S, Lee EY, Zurakowski D, Strauss KJ, Boiselle PM (2009). MDCT pulmonary angiography evaluation of pulmonary embolism in children. *AJR Am J Roentgenol*.

[17] Schoepf UJ, Goldhaber SZ, Costello P (2004). Spiral computed tomography for acute pulmonary embolism. *Circulation*.

[18] Victoria T, Mong A, Altes T, Jawad AF, Hernandez A, Gonzalez L (2009). Evaluation of pulmonary embolism in a pediatric population with high clinical suspicion. *Pediatr Radiol*.

[19] Babyn PS, Gahunia HK, Massicotte P (2005). Pulmonary thromboembolism in children. *Pediatr Radiol*.

[20] Goldhaber SZ (2002). Echocardiography in the Management of Pulmonary Embolism. *Ann Intern Med*.

[21] Mets EJ, McLynn RP, Grauer JN (2020). Venous thromboembolism in children undergoing surgery: incidence, risk factors and related adverse events. *World J Pediatr Surg*.

[22] Pati I, Masiello F, Piccinini V, De Fulvio L, Massari MS, De Angelis V (2025). Risk of Venous Thromboembolism in Infectious Diseases: A Literature Review. *Pathogens*.

[23] Butler MG, Oyetunji A, Manzardo AM (2020). Age Distribution, Comorbidities and Risk Factors for Thrombosis in Prader-Willi Syndrome. *Genes (Basel)*.

[24] Hotoleanu C (2020). Association between obesity and venous thromboembolism. *Med Pharm Rep*.

[25] Barceló A, Morell-Garcia D, Sanchís P, Peña-Zarza JA, Bauça JM, Piérola J (2019). Prothrombotic state in children with obstructive sleep apnea. *Sleep Med*.

[26] Silverberg D, Fish M, Lubetsky A, Rimon U, Raskin D, Greenberg G (2021). Long-term outcome after nonsurgical management of Paget-Schroetter syndrome. *J Vasc Surg Venous Lymphat Disord*.

